# Supplementary material for: International Trends in Lithium Use for Pharmacotherapy and Clinical Correlates in Bipolar Disorder: A Scoping Review
Source: Brain Sci. 2024 Jan 20;14(1):102. doi: 10.3390/brainsci14010102 (PMC10813799; doi:10.3390/brainsci14010102)
Supplement: Supplementary file 1 [file brainsci-14-00102-s001.zip › brainsci-2805506-supplementary.pdf]

Supplementary Table S1. Search Strategy.

Search Number	Search String
1	Bipolar Disorder/
2	(Affective Psychosis, Bipolar or Bipolar Disorder Type 1 or Bipolar Disorder Type 2 or Bipolar Mood Disorder or Depression, Bipolar or Manic Depression or Manic Disorder or Manic-Depressive Psychosis or Psychoses, Manic-Depressive or Psychosis, Manic-Depressive or Type 1 Bipolar Disorder or Type 2 Bipolar Disorder).mp.
3	1 or 2
4	Lithium.mp. or Lithium Carbonate/or Lithium/or Lithium Compounds/or Lithium Chloride/
5	Mood stabili*.mp.
6	4 or 5
7	Pharmacoepidemiology.mp. or Pharmacoepidemiology/
8	Epidemiology.mp. or Epidemiology/
9	Epidemiologic Studies.mp. or Epidemiologic Studies/
10	Epidemiological Monitoring.mp. or Epidemiological Monitoring/
11	Drug Utilization/or Drug Prescriptions/or prescription pattern*.mp.
12	7 or 8 or 9 or 10 or 11
13	3 and 6 and 12
14	limit 13 to english language
